# Supplementary material for: Neutral Polysaccharide from the Leaves of Pseuderanthemum carruthersii: Presence of 3-O-Methyl Galactose and Anti-Inflammatory Activity in LPS-Stimulated RAW 264.7 Cells
Source: Polymers (Basel). 2019 Jul 22;11(7):1219. doi: 10.3390/polym11071219 (PMC6680566; doi:10.3390/polym11071219)
Supplement: Supplementary file 1 [file polymers-11-01219-s001.pdf]

SUPPLEMENTARY MATERIAL

Neutral Polysaccharide from the Leaves of *Pseuderanthemum carruthersii:* Presence of 3-O-Methyl Galactose and Anti-Inflammatory Activity in LPS-Stimulated RAW 264.7 Cells

Vo Hoai Bac [1,2,3,*], Berit Smestad Paulsen [2], Le Van Truong [1,3], Andreas Koschella [4], Tat Cuong Trinh [5], Christian Winther Wold [2], Suthajini Yogarajah [2] and Thomas Heinze [4]

[1] Institute of Biotechnology, Vietnam Academy of Science and Technology, 18 Hoang Quoc Viet, Hanoi Vietnam
[2] Department of Pharmacy, Section of Pharmaceutical Chemistry, University of Oslo, 0316 Oslo, Norway
[3] Graduate University of Science and Technology, Vietnam Academy of Science and Technology, 18 Hoang Quoc Viet, Hanoi, Vietnam
[4] Friedrich Schiller University of Jena, Institute for Organic Chemistry and Macromolecular Chemistry, Center of Excellence for Polysaccharide Research, Humboldtstrasse, D-07743 Jena, Germany
[5] Key Laboratory for Enzyme and Protein Technology, Hanoi University of Science, Hanoi, Vietnam
* Correspondence: vhbac@ibt.ac.vn; Tel.:+84-2438360853; Fax:+84-2438363144

The mass spectrum of 3-O-Methyl Galactose

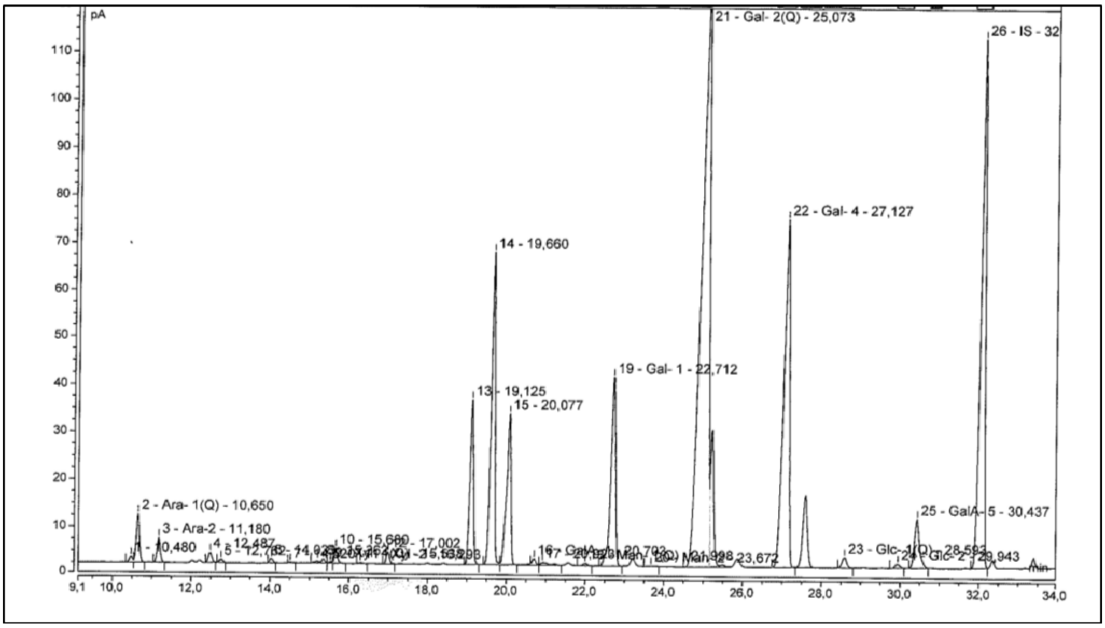

Figure S1. This trace is the GC trace after methanolysisof **PCA1** and the three peaks 13, 14 and 15 are representing the TMS derivatives of the methyl-glycoside of 3-O-methylgalactose.

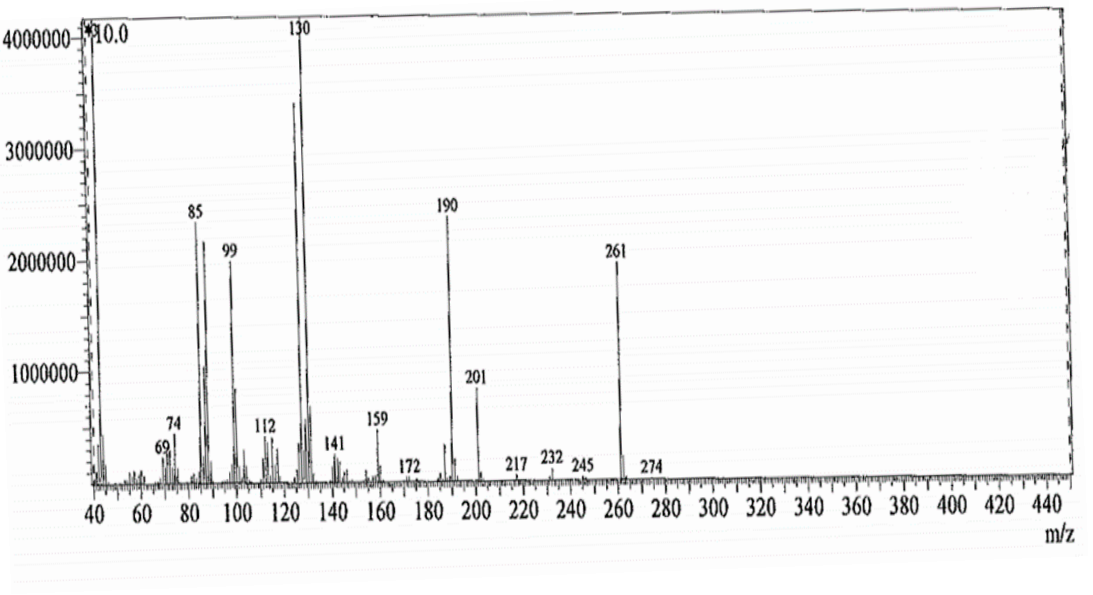

Figure S2. GC-MS of **PCA1** for identity 3-O-methylgalactose (primary fragments: 190, 261)

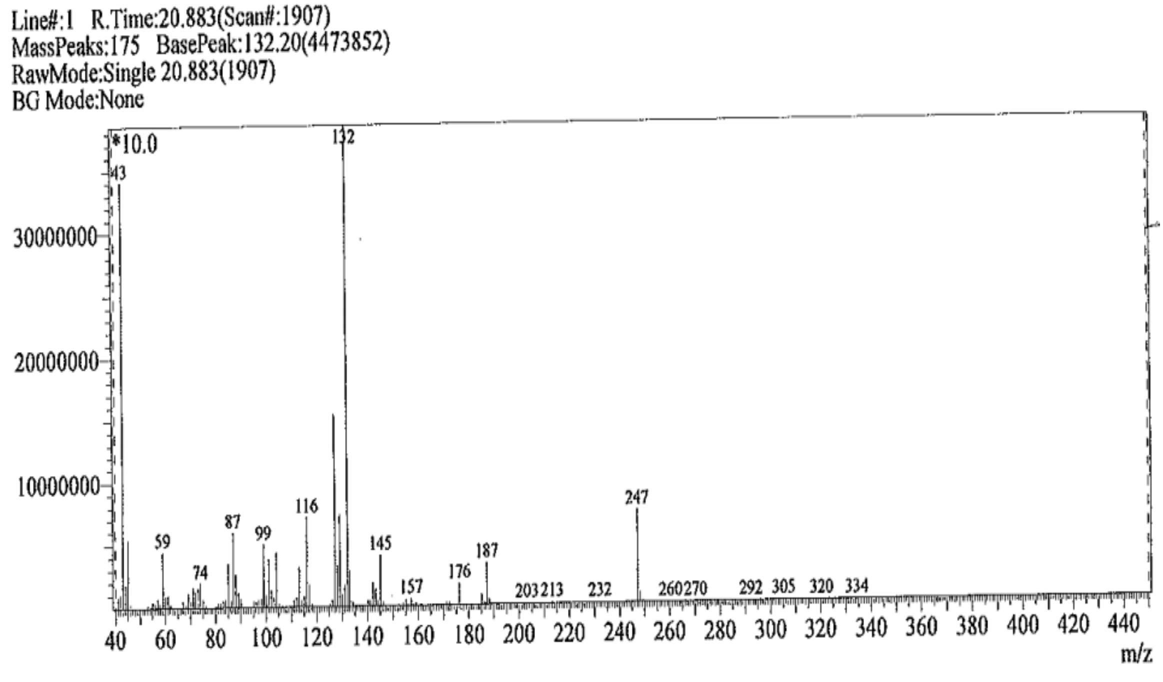

Figure S3. The mass spectrum of ethylated 3-O-methylgalactose being 1,4 linked, i.e., the ms of 1,4,5 tri-O-acetyl 2,6 di-O-ethyl 3-O-methylgalactitol

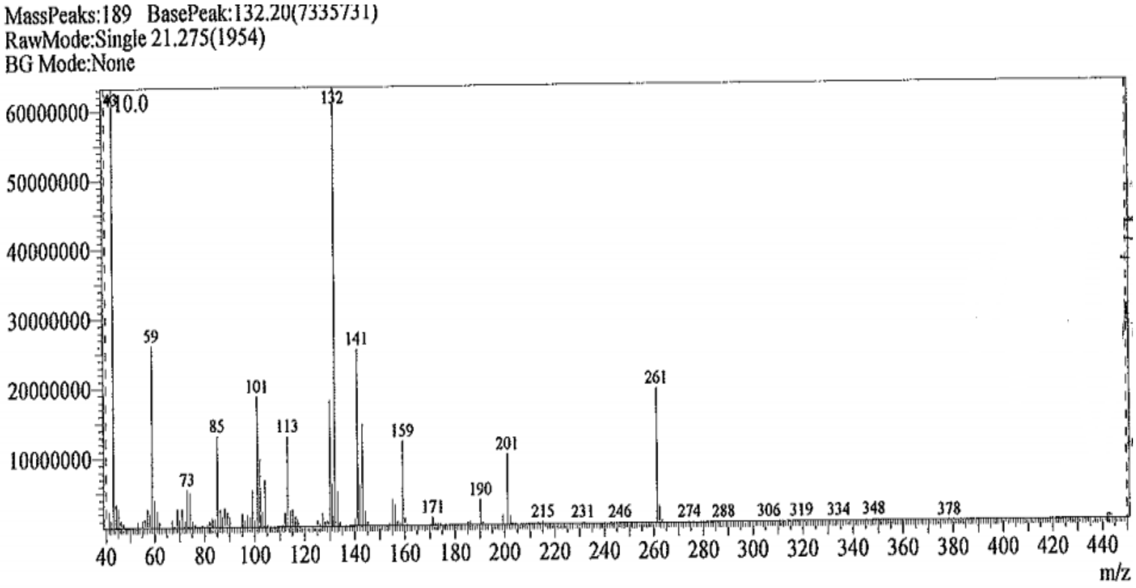

Figure S4. The mass spectrum of the ethylated 1,4 linked galactose, i.e., 1,4,5 tri-O-acetyl 2,3,6 tri-O-ethylgalactitol.

Both products obtained after ethylation, hydrolysis, reduction with sodium borodeuteride and acetylation to give the products having deuterium on C1.
